# Does allogeneic stem cell transplantation in survivors of pediatric leukemia impact regular physical activity, pulmonary function, and exercise capacity?

**DOI:** 10.1186/s40348-021-00127-7

**Published:** 2021-11-04

**Authors:** Katharina Ruf, Alaa Badran, Céline Siauw, Imme Haubitz, Paul-Gerhardt Schlegel, Helge Hebestreit, Christoph Härtel, Verena Wiegering

**Affiliations:** University Children’s Hospital, University of Würzburg, Josef-Schneider-Straße 2, 97080 Würzburg, Germany

**Keywords:** Childhood leukemia, Exercise tolerance, Physical activity, Pediatric stem cell transplantation, Exercise testing

## Abstract

**Background:**

Allogeneic hematopoietic stem cell transplantation (allo-HSCT) has improved survival in high-risk childhood leukemia but is associated with long-term sequelae such as impaired pulmonary function and reduced exercise capacity impacting quality of life.

**Methods:**

A convenience sample of 17 patients after allo-HSCT (HSCT—12 male, age 15.7±6.7 years, time after HSCT 5.3±2.8 years) underwent pulmonary function testing, echocardiography, and an incremental exercise test on a bike. Physical activity and health-related quality of life were assessed by questionnaires (7-day physical activity recall, PEDS-QL). Seventeen healthy age- and gender-matched controls served as control group (CG) for results of pulmonary function and exercise testing.

**Results:**

HSCT showed reduced pulmonary function (HSCT vs. CG: FEV1 90.5±14.0 vs. 108.0±8.7%pred; FVC 88.4±19.3 vs. 107.6±6.9%pred, DLCO 75.3±23.6 vs. 104.9±12.8%pred) and exercise capacity (VO_2_peak 89±30.8%pred, CG 98±17.5%pred; Wmax 84±21.7%pred, CG 115±22.8%pred), but no relevant cardiac dysfunction and a good quality of life (PEDS-QL mean overall score 83.3±10.7). Differences in peak oxygen uptake between groups were mostly explained by 5 adolescent patients who underwent total body irradiation for conditioning. They showed significantly reduced diffusion capacity and reduced peak oxygen uptake.

Patients reported a mean time of inactivity of 777±159min/day, moderate activity of 110±107 min/day, hard activity of 35±36 min/day, and very hard activity of 23±22 min/day. A higher amount of inactivity was associated with a lower peak oxygen uptake (correlation coefficient tau −0.48, *p*=0.023).

**Conclusions:**

This pilot study shows that although patients after allo-HSCT reported a good quality of life, regular physical activity and exercise capacity are reduced in survivors of stem cell transplantation, especially in adolescents who are treated with total body irradiation for conditioning. Factors hindering regular physical activity need to be identified and exercise counseling should be part of follow-up visits in these patients.

## Background

Allogeneic hematopoietic stem cell transplantation (allo-HSCT) has become an established treatment in pediatric high-risk leukemia to improve survival. With enhanced procedures over the last decades, the long-term survival after allo-HSCT has increased to 50–80% [1]; however, allo-HSCT is related to treatment-associated morbidity and mortality [2]. Therefore, considering long-term effects of allo-HSCT is crucial in the care for these patients. Possible and often reported sequelae of allo-HSCT are obesity, endocrine abnormalities, infertility, acute, and chronic graft-versus-host disease (GVHD), secondary malignancies, and cardiac and pulmonary dysfunction [2, 3]. Pulmonary issues account for quite a number of complications throughout the transplantation process but also thereafter [4]. Little is known, though, about exercise capacity; data so far suggest impaired exercise capacity among survivors of acute lymphoblastic leukemia in general and even a higher rate of impairment among those who underwent allo-HSCT [1, 5–8].

Exercise capacity and regular physical activity are surrogate markers for the risk of developing cardiovascular disease in both, healthy populations and patients [9]. Further, survivors of allo-HSCT are at risk for obesity, osteopenia, and insulin resistance, especially due to long-term application of corticosteroids [3]. Next to all physical side-effects of allo-HSCT, inactivity and reduced physical activity seem to play a role in this context: overprotective parents as well as a habit of inactivity evolving during long hospital stays may contribute to a persisting inactivity pattern [10]. It has been reported that especially obese survivors spent more time in sedentary and light activity [11], entering a vicious circle of cardiopulmonary but also muscular deconditioning resulting in even lower exercise capacity. Studies demonstrate, though, that regular physical activity can improve the cardiovascular risk profile in pediatric cancer survivors [12] and regular exercise training during and after cancer treatment positively affects body composition, flexibility, cardiorespiratory fitness, muscle strength, and health-related quality of life [13]. Finally, exercise capacity and physical activity are closely related to health-related quality of life in healthy children [14] but data suggests that this applies even more to survivors of childhood cancer [8, 15, 16].

The aim of this study was to determine exercise capacity, physical activity, cardiac and pulmonary function, and quality of life in survivors of leukemia undergoing allo-HSCT in childhood or adolescence. To our knowledge, this is the first study to incorporate all these aspects in one cohort of survivors of pediatric leukemia who underwent allo-HSCT.

## Methods

### Population/ethics

Between 2005 and 2015, 131 patients underwent HSCT in our center. Of the survivors of this cohort, 42 underwent allogeneic HSCT. Inclusion criteria for our study were (1) age >6 years at exercise testing, (2) allogeneic transplantation due to acute leukemia or accelerated phase of a chronic leukemia before the age of 18 years, (3) living in Germany, and (4) physical and intellectual capability to perform an exercise test on a cycle ergometer. Procedures and equipment of the study were explained and demonstrated, and verbal assent and written informed consent were obtained from the participants and their legal guardians if applicable. Of 36 patients eligible to participate in the study, 17 consented to take part.

Between July and December 2016, participants came to our center for one study visit, at which participants underwent pulmonary function and cardiopulmonary exercise testing and completed activity and quality of life questionnaires (see further down for details). Participants were all aged <18 years at allo-HSCT and 7 to 25 years of age at the time of the study. The study was approved by the local ethics committee of the University of Würzburg on October 20, 2015 (Vote number 167/15).

The results of 17 age- and gender-matched healthy individuals from previous studies in our hospital served as control groups [17, 18]. Whereas pulmonary function and exercise testing had been performed using the same equipment, unfortunately, no data on physical activity and quality of life was available for these healthy individuals.

### Pulmonary function testing

At study visit, height and weight were measured. Data of pulmonary function and lung volumes were collected by spirometry and body plethysmography according to current standards [19]. Diffusion capacity of the lung for carbon monoxide (DLCO) was assessed by single-breath-technique (MasterScope Spirometer and Master Screen Body, Jaeger/CareFusion, Höchberg); in all leukemia participants, DLCO was corrected for hemoglobin. Participants in the control group were derived from former studies: as we did not draw blood in those studies, hemoglobin was calculated according to existing normal values [20, 21] and DLCO corrected for this hemoglobin value. Forced expiratory volume in one second (FEV1), forced vital capacity (FVC), and effective resistance (Reff) were reported as %predicted [22]. Residual volume (RV) was expressed in % of total lung capacity (TLC) and reported as %predicted [22] as was DLCO [19]. Values below 80% for DLCO, FEV1, and FVC were considered abnormal, as were Reff or a RV%TLC above 120% [23].

### Echocardiography

All participants with HSCT undergo yearly echocardiographic check-ups in our center. For this study, the echocardiography closest to the date of the exercise test was chosen. All studies were performed by an experienced pediatric cardiologist with an EPIQ 5 Ultrasound system (Philips, Andover, MA, USA) using a 5-MHz transducer. The following parameters were assessed using M-Mode in a parasternal long axis: shortening fraction (SF) and ejection fraction (EF) calculated with the Teichholz formula. Further measures included intraventricular septum in diastole (IVSd), left ventricular end-systolic (LVESd) and diastolic (LVEDd) diameter, and left ventricular posterior wall in diastole (LVPWd). For diastolic assessment, the following parameters were measured on lateral mitral annulus: Early diastolic inflow velocity (peak mitral flow velocity during early ventricular diastole—E wave), peak mitral flow velocity during atrial contraction (A wave) and E/A ratio, peak early diastolic annular velocity (E’), and peak late diastolic annular velocity (A’). From these data, E/E’, the ratio of early mitral inflow to peak early diastolic annular velocity, was calculated. To evaluate the right ventricle, tricuspid annular planar systolic excursion (TAPSE) was measured using an M-mode through the lateral tricuspid annulus. Left ventricular function was further evaluated by using global longitudinal strain imaging. *Z*-scores were calculated and all data was compared to normative data [24].

### Exercise testing and assessment of physical activity

After familiarizing the patients with the cycle ergometer (Ergoselect 2000K, Ergoline, Bitz, Germany) and the gas sampling equipment, an incremental exercise test was performed up to volitional fatigue according to the Godfrey protocol [25]. Work rate was set depending on the height of the patient: patients with a height between 120 and 150 cm started with 15 W and patients taller than 150 cm started with 20 W. Work load was increased minute-by-minute by 15 W or 20 W, respectively. Maximal work load (maximal Watt achieved) was determined as the highest work rate performed for 1 min and expressed in % predicted [25] and W/kg bodyweight. During the exercise test, ventilation and gas exchange data were recorded breath-by-breath using a metabolic cart and averaged every 15 s (Vmax Encore metabolic cart system, Sensormedics, Yorba Linda, USA). Peak oxygen uptake (VO2peak) was taken as the highest oxygen uptake over three consecutive 10-s intervals during the test and expressed in % predicted [26] as well as ml/kg bodyweight.

The 7-day physical activity recall (7D-PAR) [27] is an activity questionnaire that has been evaluated by our group in the past [28]. Validation via test-retest analysis has been done showing correlation coefficients ranging from *r*=0.75 to *r* =0.85. Activity measurement was available only for the patient group.

### Assessment of health-related quality of life

Health-related quality of life (HRQL) was assessed by the Pediatric Quality of Life Inventory™ (PedsQL 4.0) and the additional Stem Cell Transplant Module. The concept of HRQL has been widely established as a measurement tool of patient-relevant burden of disease in various chronic conditions [29]. It is a multidimensional concept that incorporates measures of physical symptoms, functional status, and disease impact on psychological and social functioning. The PedsQL measures health-related quality of life in various dimensions (social, physical, school/work and emotional functioning) in healthy children and those with acute and chronic health conditions [30]. For various chronic conditions, own modules have been developed: The PedsQL 3.0 Stem cell Transplant Module is composed of 41 items comprising 8 dimensions (Pain and hurt, fatigue, nausea, worry/anxiety about the treatment, nutritional problems, thinking/remembering, communication about disease/treatment, other complaints, especially cGVHD-related). Item responses are rated on a 5-point Likert scale ranging from never to almost always. It has been validated in patients aged 2 to 18 years [31]. Separate questionnaires are available for several age groups (2–4 years, 5–7 years, 8–12 years, and 13–18 years). In all age groups, separate questionnaires were handed out to the patients and the parents to assess self-perception and that of others. The German version was employed in all patients and parents as all were German native speakers.

Further, we used the classical criteria for definition of acute and chronic graft versus host disease (GvHD) for consistency [32, 33]. According to the criteria defined by Shulman et al., chronic GvHD was defined as any GvHD after day 100 (excluding late de novo acute GvHD after DLI) and classified as limited (localized skin involvement and/or hepatic dysfunction) or extensive (all other forms) [33].

### Statistics

Patients’ characteristics and results of all tests are expressed as mean and standard deviation. Differences between groups were assessed using *t*-tests or Mann-Whitney *U* tests depending on normal distribution. Likewise, correlation analysis was performed with the help of Kendall tau or Pearson correlation coefficient depending on normal distribution. An exploratory ANOVA was calculated with VO_2_peak as dependent variable, effect size was expressed as eta square. Results with a *p*-value <0.05 (CI 95%) were considered to be significant.

## Results

Seventeen pediatric and adolescent patients who underwent allo-HSCT for leukemia (47% of the eligible cohort) and 17 healthy control participants were included in the study. Participants’ characteristics are presented in Table 1. The mean time after transplantation at the point of study was 5.3 years (standard deviation 2.8 years).

**Table 1:** Participants’ characteristics

	Patients *n*=17	Control population *n*=17	*P*-value
Male (*n* (%))	12 (70%)	11 (65%)	
Age (years)	15.7±6.7	15.6±7.1	0.92
Weight (kg)	48.0±17.1	47.4 ±19.3	**0.02**
Height (cm)	152.1±21.1	153.7 ±21.0	0.20
FEV1 (%pred) z-score	90.5 ±14.0 −0.44 (0.9)	108.0±8.7 0.45 (0.9)	**0.002**
FVC (%pred) *z*-score	88.4±19.3 -0.45 (0.7)	107.6±6.9 0.59 (0.9)	**0.001**
Reff (%pred)	88.8±34.3	78.3±36.1	0.39
RV/TLC(%pred) *z*-score	99.4±25.6 0.84 (0.7)	107.0±31.7 0.84 (1.76)	0.53
DLCO (%pred) *z*-score	75.3±23.6	104.9±12.8 0.27 (0.86)	**0.005**

All data are mean±standard deviation

Of the 17 patients who underwent allo-HSCT, 11 were treated for acute lymphatic leukemia (ALL), 5 for acute myeloic leukemia (AML), and 1 for chronic myeloic leukemia (CML) in an acute phase. Conditioning for allo-HSCT was dependent on the diagnosis and the age of the patients. All patients with ALL received total body irradiation, all patients with AML received cyclophosphamide, and of these, 4 were also treated with busulfan and 1 with total body irradiation (this was an individual therapeutic approach as this patient showed a chemotherapy resistant course and was transplanted in partial response). The patient with CML was conditioned with cyclophosphamide and total body irradiation. Of the 17 patients, 11 received peripheral blood stem cells, 6 bone marrow as graft. After transplantation, 7 patients showed acute GvHD grade 1, 4 patients grade 2, and one patient suffered from acute GvHD grade 3. Six patients (38%) showed signs of chronic, but—at the time of this study—resolved GvHD. None showed a chronic GvHD higher than grade 2; especially no severe pulmonary GvHD was seen in any patient.

During transplantation, none of the patients suffered from severe pulmonary or cardiac complications such as pneumonia, sepsis, or systemic fungal infection.

### Pulmonary function

Compared to healthy controls, patients after allo-HSCT showed significantly lower FEV1%pred and FVC%pred as well as a lower diffusion capacity for carbon monoxide (see Table 1); still, with the exception of DLCO, values were still within the normal range. No differences were found in specific resistance and residual volume. When comparing test results before and after allo-HSCT in the patient group, no significant differences were found (data not shown).

### Echocardiography

The patients’ results of the echocardiography are presented in Table 2. We did not find significant cardiac dysfunction in our patients. Parameters for right and left ventricular function were normal as well as global parameters like shortening fraction and ejection fraction.

**Table 2:** Echocardiographic data

Parameter	Value	*z*-score
IVSd (cm)	0.7±0.15	0.37±0.57
IVSs (cm)	1.0±0.20	0.18±0.81
LVEDd (mm)	43.8±4.7	0.05±0.86
LVEDs (mm)	29.1±2.8	0.59±0.84
LVPWd (mm)	8.0±1.6	1.31±0.55
LVPWs (mm)	10.6±2.2	−0.76± 0.8
FS (%)	33.1±2.9	
EF (%)	61.4±4.4	
E wave (mm/s)	95.4±17.5	
A wave (mm/s)	54.7±9.6	
E/A	1.70±0.4	
E'Lat (mm/s)	12.5±2.3	
E/E′	7.8±1.6	
TAPSE (mm)	22.7±0.4	
Left ventricular global long strain (%)	−20.8±2.6	

All data are mean±standard deviation

### Exercise testing

**Fig. 1:**
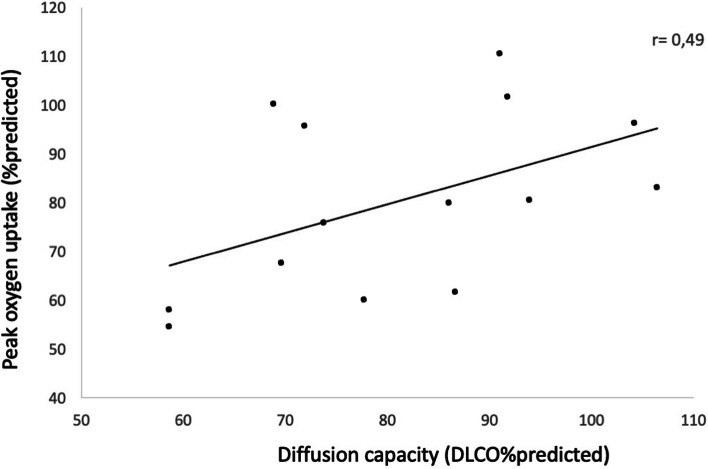
Relationship of diffusion capacity (in %predicted) and peak oxygen uptake (in %predicted). On the *y*-axis, peak oxygen uptake is presented; on the *x*-axis diffusion capacity, both expressed in %predicted. The correlation between the two is illustrated by the regression line; for further information, the Pearson correlation coefficient is included in this figure

All participants reached a maximal effort during the exercise test with a heart rate >85% of the predicted maximal heart rate and an RQ of >1.1 [34]. No significant differences were seen in peak heart rate or RQ between the patients and healthy controls. Patients after HSCT showed reduced exercise capacity as demonstrated in Table 3. This finding is mainly attributed to 5 patients (30% of the cohort), who showed an abnormal response to exercise with 55 to 67% of predicted peak oxygen uptake. All 5 patients had normal cardiac function (see above) but showed decreased diffusion capacity (4 of these 5 individuals showed a DLCO of 58–78%predicted, see Fig. 1). Four of these patients received HSCT for ALL, one for AML; all were adolescents at the time of allo-HSCT and were conditioned with TBI.

**Table 3:** Results of exercise testing and physical activity

	Patients	Control population	*P*-value
**Exercise testing**			
Heart rate at rest (bpm)	91±14	98±16	0.33
Peak heart rate (bpm)	186±10	189±11	0.55
Peak oxygen saturation (%)	98±1.5	99±1.1	0.09
Maximal work load (%predicted)	84±21.7	115±22.8	**0.001**
Maximal work load (W/kg)	2.4±0.7	3.4±0.7	**0.001**
Peak oxygen uptake (%predicted)	89±30.8	98±17.5	0.12
Peak oxygen uptake (ml/min/kg)	39.1±7.9	46.2±9.5	**0.04**
Respiratory exchange ration	1.12±0.16	1.11±0.11	0.99
Peak tidal volume (l)	1.5±0.7	1.5±0.7	0.95
**Physical activity**			
7D-PAR			
Inactivity (min/day)	777±159	Not assessed	
Moderate activity (min/day)	110±107	Not assessed	
Hard activity (min/day)	35±36	Not assessed	
Very hard activity (min/day)	23±22	Not assessed	

All data are mean±standard deviation

A comparison of patients who received TBI in contrast to those who received chemotherapy alone showed no significant differences in pulmonary function (FEV1, FVC, TLCOC) nor in exercise capacity (see Table 4). Due to the small sample size, calculating ANOVAs to correct for various confounders was difficult. An exploratory analysis with VO_2_peak%predicted as dependent variable showed that age (*p*=0.030, eta square 0.29) but not total body irradiation (*p*=0.211) influenced peak oxygen uptake.

**Table 4:** Comparison of patients receiving TBI and chemotherapy alone

	TBI *n*=12	Chemotherapy alone *n*=5	*P*-value
Age (years)	16.4±7.3	13.2±5.6	0.40
Weight (kg)	47.0±16.9	50.4 ±19.3	0.72
Height (cm)	154.5±22.4	146.3 ±18.5	0.48
FEV1 (%pred)	92.5 ±11.1	98.9±10.9	0.292
FVC (%pred)	94.3±8.0	93.5±9.7	0.33
RV/TLC(%pred)	114.6±19.1	115.8.0±37.8	0.93
DLCO (%pred)	81.1±16.6	82.4±12.0	0.91
Peak heart rate (bpm)	186±11	192±3	0.15
Maximal work load (%predicted)	96±58	96±21	0.98
Maximal work load (W/kg)	2.9±1.3	2.1±0.4	0.28
Peak oxygen uptake (%predicted)	81±20	107±45	0.12
Peak oxygen uptake (ml/min/kg)	40.0±6.8	36.9±10.8	0.48

All data are mean±standard deviation

### Physical activity

The 7D-PAR revealed that patients were inactive for 777±159 min, engaged in moderate activity with a mean time of 110±107min/day, in hard activity 35±36 min/day and 23±22 minutes/day in very hard activity. We found a moderate negative correlation between peak oxygen uptake and inactivity (tau =−0.48, *p*=0.023).

### Quality of life

Overall, patients showed an acceptable quality of life in both, the PedsQL as well as in the transplant module (for patient-reported results, see Table 5). No significant differences were found between the answers of parents and patients (data not shown) in the transplant module nor in the overall PedsQL score. No significant correlations were found between the parameters of the PedsQL and parameters of exercise testing or regular physical activity.

**Table 5:** Measurement of quality of life

Domains	Mean±standard deviation (range)
PEDSQL 4.0	
Physical functioning	89.3±11.8 (62.5–100)
Emotional functioning	77.2±20.3 (25–100)
Social functioning	88.8±12.5 (55–100)
School/work functioning	80.9±9.9 (65–100)
Overall score	84.7±10.8 (56.5–100)
Transplant module	
Pain and hurt	80.5±19.3 (37.5–100)
Fatigue	76.5±15.8 (40.0–100)
Nausea	91.8±13.4 (56.3–100)
Worry/anxiety about disease/treatment	83.6±12.3 (58.3–97.5)
Nutritional problems	83.8±16.5 (35–100)
Thinking/remembering	72.7±19.9 (37.5–100)
Communication about disease/treatment	84.4±20.2 (41.7–100)
Other complaints—specifically cGVHD-related problems	92.5±8.1 (75–100)
Overall	83.3±10.7 (55.9–98.1)

All data are mean±standard deviation

## Discussion

The main finding in this pilot study is that—after allo-HSCT for pediatric leukemia—children, adolescents and young adults show reduced exercise capacity and pulmonary function compared to healthy controls particularly after receiving total body irradiation in adolescence while showing an acceptable health-related quality of life.

Especially impairments in diffusion capacity are evident compared to healthy individuals. A closer look at the cohort reveals that those with reduced diffusion capacity are also those with lower exercise capacity. Interestingly, these patients were all conditioned with TBI in adolescence. They seem to be a vulnerable group which warrants a thorough follow-up due to an increased risk of posttransplant toxicity. Our sample size is small, though, and we were not able to calculate analysis of variance, which would allow to account for further confounders. Still, impaired pulmonary function seems to limit exercise capacity, which has been demonstrated in other lung diseases such as cystic fibrosis [35] or bronchopulmonary dysplasia [36] and has also been described in patients after allo-HSCT [6, 37, 38]. The incidence of post-HSCT lung disease has been 25% in a retrospect analysis in our hospital over 11 years [39] and studies analyzing pulmonary function after allo-HSCT equally demonstrate that pulmonary impairment after allo-HSCT is common [40]; however, severe pulmonary impairment is rare [41]. Further studies with larger cohorts are needed to differentiate the impact of chemotherapy, conditioning regime and allo-HSCT on pulmonary function.

In our cohort, cardiac dysfunction does not seem to affect exercise capacity, as cardiac function is not impaired in our patients after a mean of 5 years after allo-HSCT. In the abovementioned retrospect analysis in our hospital on outcomes after allo-HSCT over a time of 11 years, only 6% developed cardiomyopathy, showing that cardiac sequelae are rather rare compared to pulmonary impairment [39].

Overall, patients in our study show a slightly reduced exercise capacity compared to healthy controls. Limitations in peak workload in our cohort were comparable to other cohorts of children after allo-HSCT [6, 37, 38]. Possible reasons for such limitations are impaired pulmonary function, physical inactivity, pain, fatigue, and an unproven fear on exercise by patients and their parents. As mentioned above, decreased lung diffusion capacity is linked to impaired exercise capacity [40].

Since nowadays, the positive effects of regular physical activity are well-known, exercise promotion is crucial in the counseling of children with chronic conditions. Regular physical activity can prevent osteoporosis and cardiovascular and metabolic disease in this population as increased exercise capacity improves cardiac output and capillary density in the muscles [42] and enhances quality of life after allo-HSCT [43–45]. Measurement of physical activity has increasingly gained importance over the last years, and especially inactivity is associated with health issues and limitations in daily life [46, 47]. Prolonged hospital stays may lead to less promotion of physical activity by the family and consecutively to physical deconditioning. When comparing patients of our study to a cohort of patients with cystic fibrosis, we found a similar amount of moderate activity, but reduced engagement in hard and very hard activity, although exercise capacity in both groups seems comparable with a mean peak oxygen uptake of 89%predicted in the cystic fibrosis cohort [28] and this cohort. Compared to a cohort of children suffering from insulin-dependent diabetes, our cohort shows significantly lower activity times: the study including children with insulin-dependent diabetes reports at least 210 min of moderate activity (also assessed by the 7D-PAR), which is almost the double amount compared to our patients and at least 60 min of vigorous activity (the amounts hard and very hard activity were summed up in that study, still, this cohort showed at least 5 more min of hard activity per day) [48]. Screening for inactivity and possible barriers of regular physical activity is of utmost importance in leukemia survivors in order to intervene early. Research has shown that activity interventions such as supervised training or online interventions with activity guidelines decrease inactivity time and fatigue and improve exercise tolerance, HRQL, and regular physical activity [49–53].

Patients in this study showed a good overall HRQL (mean 85) which is comparable to a healthy population (mean 83) [54]. Although a relation between exercise capacity and HRQL has been postulated in the past [14, 55], we were not able to reproduce these findings in our cohort, probably due to a positive selection bias as patients in our cohort showed an almost normal exercise capacity with the exception of the 5 patients mentioned above. In a group of patients with juvenile idiopathic arthritis, HRQL reached the level of healthy children 3 years after beginning of treatment. The mean time after allo-HSCT in our cohort was more than 5 years; maybe HRQL was lower closer to allo-HSCT. Further studies are needed to see whether an exercise intervention can improve HRQL, as in former studies decreased physical functioning has been shown to impact quality of life in survivors of childhood cancer [15, 16].

### Limitations

Although this is a small pilot study, some patients in our cohort after allo-HSCT for pediatric leukemia have impaired exercise tolerance and show a reduced level of regular physical activity. We most certainly have a positive selection bias with a convenience sample of rather healthy survivors of pediatric leukemia. This results in good HRQL and with the exception of the 5 adolescents already discussed in an almost normal exercise capacity. Of 36 eligible participants, only 17 took part in this study and it can be assumed that these might be the ones interested in activity and exercise and probably are more likely engaged in regular exercise. Further, our patients were not overweight or obese, which is a common complication after treatment for pediatric leukemia. It can be expected that those who are fitter and more active are also those more willing to participate in a study analyzing exercise capacity and physical activity. This probably results in a significant selection bias. However, already in this rather healthy group, physical activity is reduced and, in some patients, we see decrements in exercise capacity as elaborated above.

In our control group, participants show pulmonary function test results that are slightly above 100% predicted; as explained above, with the exception of DLCO, we observed lower pulmonary function test results in the patient group which though are still within the range of normal values. The question whether this difference is due to a positive selection bias in the control group or to reduced pulmonary function in the patient group remains unsolved; it can be assumed that allo-HSCT and condition has a negative effect on pulmonary function [39].

We fear that our rather healthy patient group may represent the tip of the iceberg with regard to negative effects of allo-HSCT and survivors with more, especially pulmonary impairments of allo-HSCT show worse exercise capacity and higher levels of inactivity which consecutively result in lower HRQL and further sequelae of inactivity.

To exactly measure physical activity, accelerometers have become the gold standard. Accelerometers are little devices that generate data from acceleration during movement which is then transformed into activity counts via predefined algorithms. Consecutively, the time spent in different activity categories can be derived by using these counts. However, accelerometry is time-consuming and expensive whereas validated questionnaires can be quickly filled in and may therefore serve as an everyday tool to assess activity during a follow-up visit, which is why we used them instead of accelerometry in this study.

One further drawback of this study is the missing data on HRQL and physical activity in the control group. In the discussion we were able, though, to compare our data with that of former studies on children with chronic conditions.

We are aware that especially long-term outcomes after allo-HSCT are relevant which we cannot provide since this study was designed as a pilot study. Further, this study was done in a small convenience sample and results may not be generalizable especially as individual pulmonary function and conditioning for HSCT have relevant impact on the patient’s health, outcome, and exercise capacity. Nevertheless, already in this small sample, we see possible threats for the long-term outcome of these patients, namely a high amount of inactivity and reduced exercise capacity. Therefore, further research is needed to clarify the course of pulmonary function, quality of life, physical activity, and exercise capacity over time in a larger sample and identify possible approaches for targeted intervention to improve patient outcome.

## Conclusions

In our convenience sample, patients show a good HRQL; however, pulmonary impairment is present and contributes to impaired exercise capacity. This is particularly the case in adolescents who underwent TBI for conditioning warranting special attention to this issue in future studies. Further studies are needed to analyze the complex interaction between cancer therapy and the mode of conditioning on the one hand, and pulmonary and cardiac impairment and its impact on exercise capacity and regular physical activity on the other hand. As inactivity was associated with a lower peak oxygen uptake in this study, regular exercise counseling and integration of these patients in long-term follow up programs such as “Care for Caya” seem crucial to prevent negative effects of an inactive lifestyle [56].

## Data Availability

The datasets used and analyzed in this study are available from the corresponding author upon reasonable request.

## Pulmonary function

DLCO: Diffusion capacity of the lung for carbon monoxide
FEV1: Forced expiratory volume in one second
FVC: Forced vital capacity
Reff: Effective resistance
RV/TLC: Residual volume in % of total lung capacity
%pred: Percent predicted

## Cardiac function

IVSd (cm): Intraventricular septum in diastole
IVSs (cm): Intraventricular septum in systole
LVEDd (mm): Left ventricular end-diastolic diameter
LVEDs (mm): Left ventricular end-systolic diameter
LVPWd (mm): Left ventricular posterior wall diameter in diastole
LVPWs (mm): Left ventricular posterior wall diameter in systole
FS: Shortening fraction
EF: Ejection fraction
E/E’: Ratio of early mitral inflow to peak diastolic annular velocity
TAPSE (mm): Tricuspid annular planar systolic excursion

## Exercise testing and physical activity

7D-PAR: Seven-day physical activity recall questionnaire
LRC: Lipid research clinics questionnaire
RQ: Respiratory quotient
VO_2_peak: Peak oxygen uptake
Wmax: Peak work load

## Further abbreviations

ALL: Acute lymphatic leukemia
AML: Acute myeloic leukemia
CML: Chronic myeloic leukemia
DLI: Donor lymphocyte infusion
GvHD: Graft versus host disease
HSCT: Hematopoetic stem cell transplantation
HRQL: Health-related quality of life
PEDS-QL: Pediatric quality of life inventory questionnaire
TBI: Total body irradiation
